# Supplementary material for: Cancer Therapy-Related Cardiac Dysfunction in Patients Treated with a Combination of an Immune Checkpoint Inhibitor and Doxorubicin
Source: Cancers (Basel). 2022 May 7;14(9):2320. doi: 10.3390/cancers14092320 (PMC9100163; doi:10.3390/cancers14092320)
Supplement: Supplementary file 1 [file cancers-14-02320-s001.zip › cancers-1678796-supplementary.pdf]

Supplementary Materials

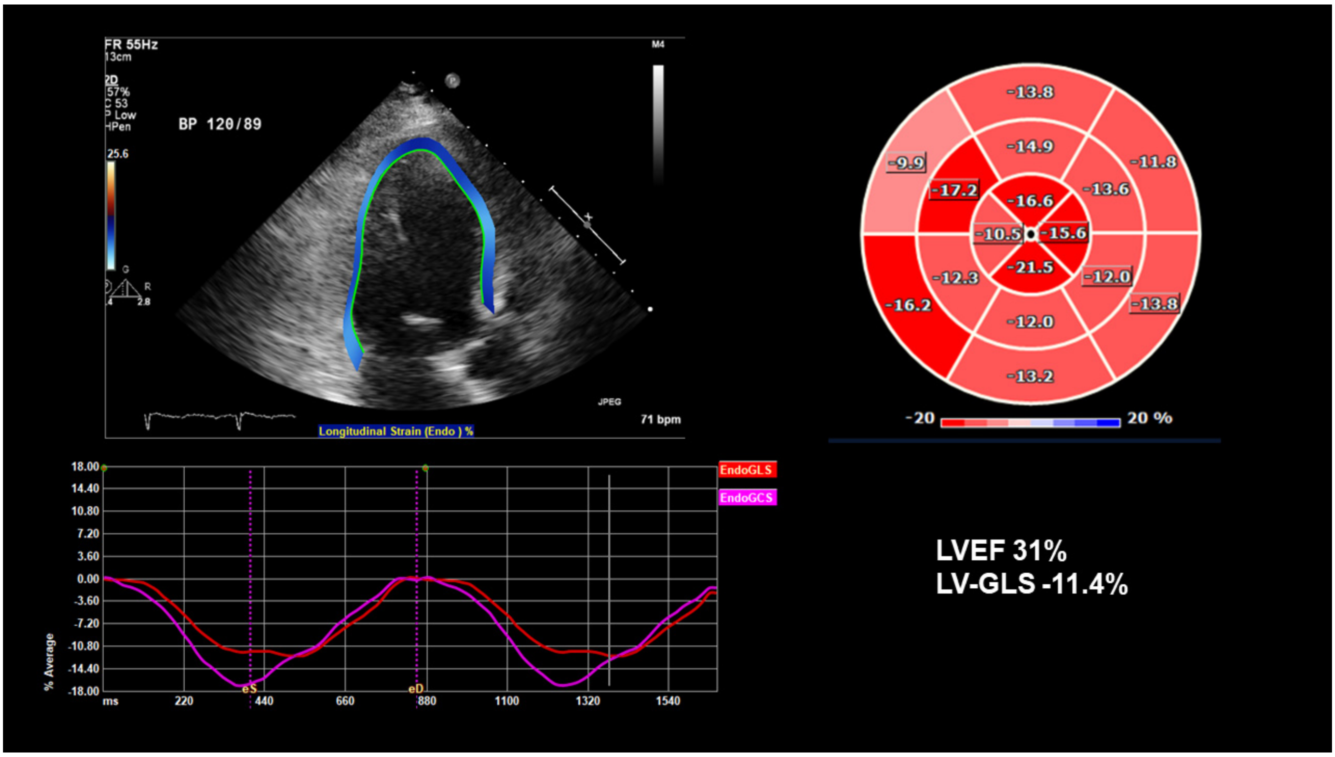

Figure S1. Representative images for assessing LVGLS.

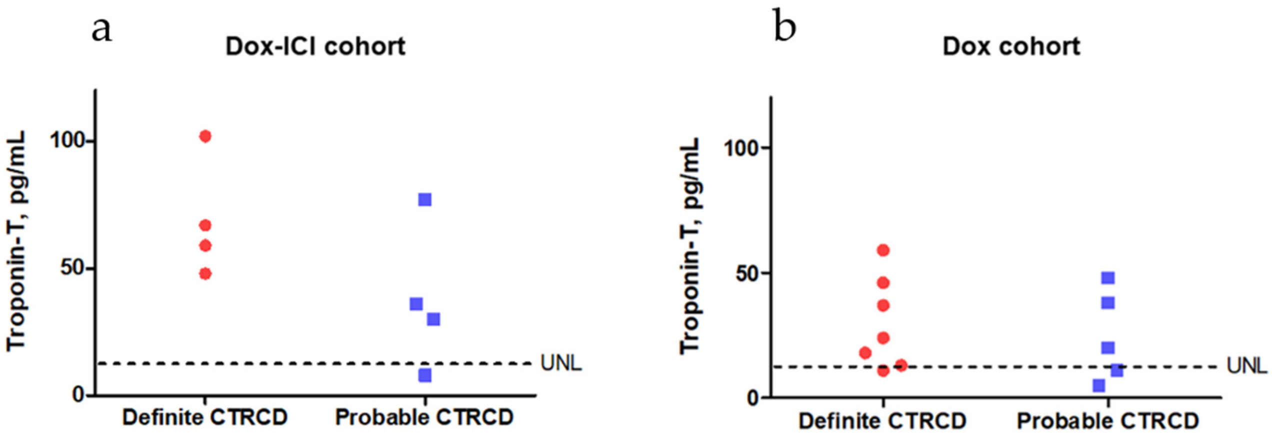

Figure S2. :(**a**) and (**b**) In both the Dox-ICI and Dox cohorts, more than half of the patients who developed probable and definite CTRCD had elevated troponin-T levels above the upper normal limit

Table S1. Change of echocardiographic parameters during serial follow up.

Parameter	Dox-ICI cohort (*n* = 22)					Dox cohort (*n* = 73)				
	Baseline	3-month	6-month	p value*	p value†	Baseline	3-month	6-month	p value*	p value†
LVEF, %	65.5 ± 5.3	66.0 ± 5.2	55.2 ± 9.0	0.691	0.003	66.6 ± 6.3	61.6 ± 9.2	59 ± 6	0.001	<0.001
LVGLS, %	−18.6 ± 1.9	−17.6 ± 4.1	−15.3 ± 3.6	0.250	0.015	−17.3 ± 2.3	−16.3 ± 2.3	−15.4 ± 3.6	0.139	<0.001

LVEF, left ventricular ejection fraction; LVGLS, left ventricular global longitudinal strain. *p for baseline vs. 3-month; †p for baseline vs. 6-month.
